# Large Variation in Listed Chargemaster Price for Total Joint Arthroplasty Among Top Orthopaedic Hospitals in the United States

**DOI:** 10.5435/JAAOSGlobal-D-23-00052

**Published:** 2023-09-07

**Authors:** Jordan R. Pollock, Matthew K. Doan, M. Lane Moore, Jack M. Haglin, Jaymeson R. Arthur, David G. Deckey, Karan A. Patel, Joshua S. Bingham

**Affiliations:** From the Mayo Clinic Alix School of Medicine, Scottsdale, AZ (Pollock, Doan, Dr. Moore, and Dr. Haglin), and the Department of Orthopedic Surgery, Mayo Clinic, Phoenix, AZ (Dr. Arthur, Dr. Deckey, Dr. Patel, and Dr. Bingham).

## Abstract

**Background:**

Chargemasters are lists of all services offered by a hospital and their associated cost. This study analyzes chargemaster data to determine price differences among different hospitals for total joint arthroplasty.

**Methods:**

In May 2020, the chargemaster data for highly rated orthopaedic hospitals were accessed, and the diagnostic-related group (DRG) codes related to primary and revision total joint arthroplasty were analyzed (DRGs 466, 467, 468, 469, and 470). The prices listed for each hospital were averaged, and descriptive statistics were calculated. Furthermore, Medicare reimbursement was collected. A subanalysis was performed to determine relationships between geographic and demographic information.

**Results:**

The median price for a major hip or knee joint arthroplasty without complications was $68,016 (range: $39,927 to $195,264). The median price of a revision of hip or knee arthroplasty without complications was $90,966 (range: $58,967 to $247,715). The cost of living in the city in which the hospitals are located was weakly correlated with procedure pricing, whereas the median income had no notable relationship to chargemaster pricing.

**Conclusion:**

The published cost of DRG codes in arthroplasty is widely variable among the top 20 US orthopaedic hospitals, with little correlation to the cost of living or median income of the area.

Orthopaedic care is highly used in the United States, accounting for over 30 million office visits in 2016.^1^ This orthopaedic care is costly, with orthopaedic surgeons collecting an estimated 3 billion dollars in 2018 from Medicare alone.^2^ Given the high prices charged for health care and the complex payor mix that exists in the United States, the actual cost of a hospitalization or procedure is often unclear until after the service has been performed.^3^ Then, the patient receives a bill based on already performed services that their insurance may or may not decide to cover.

These unexpected bills are a common complaint among patients according to various studies analyzing common complaints in various healthcare settings.^4,5,6,7^ Price transparency for medical services in the United States has been a concern for decades.^8^ However, as negotiations between insurers and healthcare systems are often kept private, it may be difficult to accurately inform patients regarding accurate medical procedure pricing. Increased price transparency for health services in the United States would improve health care in the United States by empowering patients to budget for medical expenses, apply for the financial aid at ideal times in the treatment course, and make maximally informed decisions about their medical treatment. As our aging cohort continues to increasingly use elective services, price transparency will be increasingly important to inform patient decision making and drive competition and cost containment within the elective services healthcare market.

The Centers for Medicare & Medicaid Services released a rule in 2018 to improve the price transparency of medical treatment by requiring all hospitals to publicly publish healthcare pricing information in a “chargemaster” by January 2019.^9^ A chargemaster is a compilation of prices for medical services, including common procedures such as total joint arthroplasty. This rule attempted to increase price transparency for healthcare consumers (ie, the patient) in an effort to decrease healthcare costs.^9^ This method of publishing public price information for patients, however, has been criticized for offering inaccurate and potentially misleading information regarding the true cost of medical care.^10,11^ The prices listed in chargemasters are what a hospital wants to charge a consumer for a medical service and is often inflated to assist the hospital in reimbursement negotiations.^10,12^ These charges may vary substantially and may not be an accurate representations of the actual costs.^13^

Given the large volume of elective total joint arthroplasty procedures performed in the United States and the importance of healthcare transparency for patients, we sought to investigate the variability of chargemaster data for arthroplasty in the United States. The primary purpose of this study is to assess the price variation of Medicare Severity–Diagnosis-Related Group (DRG) codes for hip and knee arthroplasty procedures using chargemaster price listings.

## Methods

The US News and World Report website ranks orthopaedic hospitals according to four major criteria: patient experience, structure, outcomes, and process/expert opinion.^14^ In an effort to control for quality of care, we chose to sample the top 20 orthopaedic hospitals, as ranked by the US News and World Report's website in May 2020.^15^ We then searched each institution's website for their published chargemaster information. Hospitals that had not yet posted this information on their websites or did not include standard pricing for various DRG codes were excluded from the analysis.

Among these chargemasters, there were five DRG codes relating to total knee arthroplasty and total hip arthroplasty. These codes were selected for further analysis in our study. The data from each chargemaster were extracted pertaining to each of these five codes (Table 1). These DRG codes included: “Revision of hip or knee arthroplasty with major complication or comorbidity (MCC)” (DRG 466), “Revision of hip or knee arthroplasty with complication or comorbidity (CC)” (DRG 467), “Revision of hip or knee arthroplasty without CC/MCC” (DRG 468), “Major hip and knee joint arthroplasty or reattachment of lower extremity with MCC or total ankle arthroplasty” (DRG 469), and “Major hip and knee joint arthroplasty or reattachment of lower extremity without MCC” (DRG 470).

**Table 1:** Orthopaedic Diagnosis-Related Group Codes and Their Respective Descriptions

DRG Code	Description
466	Revision of hip or knee arthroplasty with MCC
467	Revision of hip or knee arthroplasty W/CC
468	Revision of hip or knee arthroplasty W/O CC/MCC
469	Major hip and knee joint arthroplasty or reattachment of lower extremity W/MCC or total ankle arthroplasty
470	Major hip and knee joint arthroplasty or reattachment of lower extremity W/O MCC

DRG = diagnosis-related group, W/CC = with complications or comorbidities, W/MCC = with major complications or comorbidities, W/O CC/MCC = without complications/major complications or comorbidities

The price for each procedure was averaged across all the included hospitals, and descriptive statistics were calculated for each DRG code, including median, mean, and SD. A separate analysis was also used to examine possible correlations between prices of healthcare services and the median income and cost of living in each hospital's respective location. The US Census Bureau's 2018 Median Income report was used to find the median income of the county in which the hospital was located.^16^ A website named “bestplaces.net” and the cost-of-living database were used in May 2020 to extract the median cost of living in the corresponding county of the hospital.^17^ A linear regression analysis was used to compare the listed DRG prices with the median income and cost of living.

The accuracy of chargemaster billing information and variation was assessed by comparing the 10th and 90th percentile price for each DRG code with the degree of variation that is commonly seen in inpatient commercial claims databases. The variation commonly seen in inpatient commercial claims databases is approximately 50% to 400%.^18,19^ To provide an accurate comparison regarding chargemaster prices with actual reimbursement, the Medicare reimbursement to hospitals for all admissions for each DRG in 2018 was collected from the 2018 Inpatient Utilization and Payment Public Use File from the Center for Medicare and Medicaid Services. The mean Medicare reimbursement across all admissions was calculated for each DRG and compared with the mean chargemaster listing for each DRG. The percentage of the mean chargemaster price that was reimbursed by Medicare on average was calculated, along with the R-squared value of the correlation across the mean chargemaster price and mean Medicare reimbursement. Microsoft Excel (Microsoft) was used to perform the data analysis and extraction in this study. This study was deemed exempt from our institutional review board because hospital chargemaster data are publicly available and do not contain patient information.

## Results

Of the top 20 orthopaedic hospitals included in our study, 18 (90%) had publicly available pricing information for the chosen arthroplasty-related DRG codes in a DRG format on their websites. Of the two chargemasters that were unable to be analyzed, one hospital did not have chargemaster data publicly available, and the other hospital did not have procedure pricing listed in the DRG code format. All chargemaster data stratified by the hospital are included as a supplement. The median price for a revision of hip or knee arthroplasty with MCC (DRG 466) was $221,927 (range: $107,582 to $472,517; SD: $107,919). The median price for a revision of hip or knee arthroplasty with minor CC (DRG 467) was $117,290 (range: $68,818 to $317,806; SD: $80,118). The median price for a revision of hip or knee arthroplasty without CC (DRG 468) was $90,966 (range: $58,967 to $247,715: SD: $63,255). The median price for a primary hip or knee arthroplasty with MCC (DRG 469) was $128,290 (range: $74,588 to $324,985; SD: $79,794). The median price for a primary hip or knee joint arthroplasty without CC (DRG 470) was $68,016 (range: $39,927 to $195,264; SD: $49,745). The difference factor between 10th and 90th percentile costs was 3.2 for DRG 466, 3.6 for DRG 467, 3.6 for DRG 468, 3.6 for DRG 469, and 3.8 for DRG 470 (Table 2).

**Table 2:** Descriptive Statistics for the Five Included Diagnosis-Related Group Codes From Top 20 Orthopaedic Hospital Chargemaster Documents, Including 10th and 90th Percentile Values for Each Orthopaedic Arthroplasty Chargemaster Diagnosis-Related Group Code and the Difference Factor Between the 10th and 90th Percentile Values

DRG Code	Mean (USD)	Median (USD)	SD (USD)	Lowest Price (USD)	Highest Price (USD)	Difference Factor Between the Highest and Lowest Price	10th Percentile	90th Percentile	Difference Factor Between the 10th and 90th Percentile Price
466	$238,339	$221,927	$107,919	$107,582	$472,517	4.4	$126,273	$401,890	3.2
467	$147,960	$117,290	$80,118	$68,817	$317,806	4.6	$81,131	$292,300	3.6
468	$116,846	$90,966	$63,255	$58,967	$247,715	4.2	$63,808	$231,000	3.6
469	$154,256	$128,290	$79,794	$74,588	$324,985	4.4	$78,944	$287,482	3.6
470	$85,115	$68,016	$49,745	$39,927	$195,264	4.9	$45,690	$172,979	3.8

DRG = diagnosis-related group

**Figure 1:**
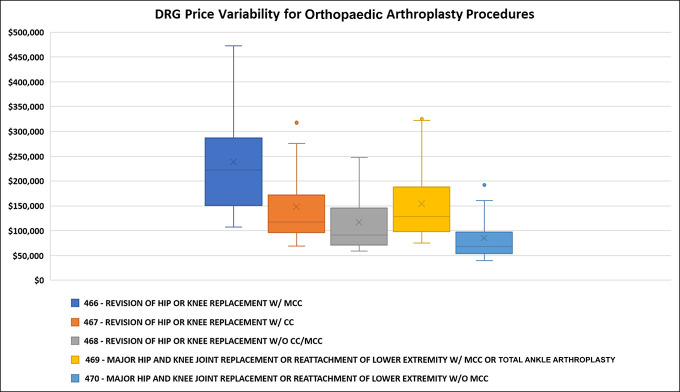
Price variation for the included orthopaedic upper extremity chargemaster diagnosis-related group (DRG) codes. W/CC = with complications or comorbidities, W/MCC = with major complications or comorbidities, W/O CC/MCC = without complications/major complications or comorbidities

**Figure 2:**
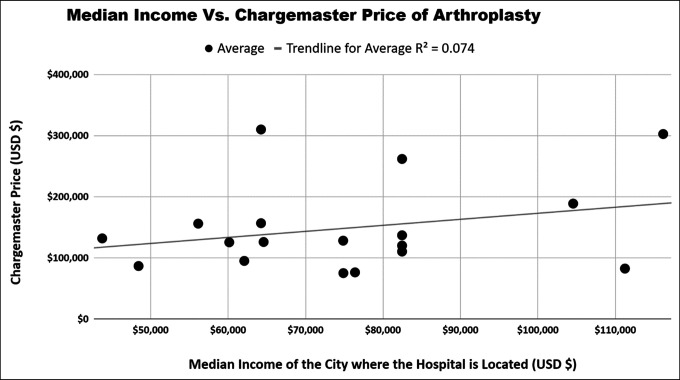
Association between the median income and average arthroplasty chargemaster price.

**Figure 3:**
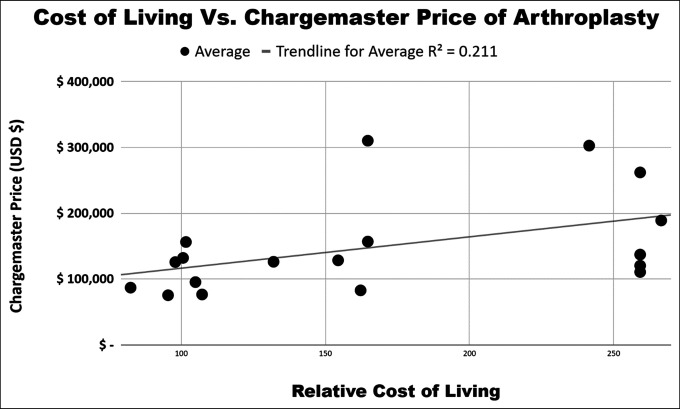
Association between the cost-of-living index and average arthroplasty DRG. DRG = diagnosis-related group

The cost of living in the city in which the hospitals are located was weakly correlated with the chargemaster price of all arthroplasty procedures averaged together (R^2^ of 0.211, *P* = 0.05). DRG 469 was the only individual procedure significantly correlated with the cost of living (R^2^ of 0.27, *P* = 0.03). We found no correlation with the median income of the county in which the hospitals are located (R^2^ = 0.07, *P* = 0.27) for all arthroplasty procedures averaged together. Individually, no DRG code had a notable association with the median income of the city in which the hospitals are located (Table 3, Figures 1–3).

**Table 3:** Relationship Between the Cost of Living and Median Income With Specific Orthopaedic Arthroplasty Chargemaster Diagnosis-Related Group Codes

	R Square	*P*
DRG 466		
Cost of living	0.20	0.07
Median income	0.04	0.45
DRG 467		
Cost of living	0.18	0.09
Median income	0.07	0.29
DRG 468		
Cost of living	0.15	0.11
Median income	0.05	0.35
DRG 469		
Cost of living	0.27	0.03^a^
Median income	0.12	0.18
DRG 470		
Cost of living	0.23	0.05
Median income	0.12	0.17
All DRG codes		
Cost of living	0.21	0.05
Median income	0.07	0.27

DRG = diagnosis-related group

a Indicates significance.

The mean chargemaster listing price exceeded the mean Medicare reimbursement to hospitals for all DRGs. The mean Medicare reimbursement to hospitals in 2018 was $36,123 for DRG 466, $21,922 for DRG 467, $17,174 for DRG 468, $19,734 for DRG 469, and $11,828 for DRG 470. When comparing this mean reimbursement with mean chargemaster price, Medicare reimbursement accounted for 15.2%,14.8%, 14.7%, 12.8%, and 13.9% of the mean chargemaster price for DRGs 466, 467, 468, 469, and 470, respectively. The Medicare reimbursement and the listing price for each DRG across all included hospitals had an R-squared value of 0.15 for DRG 466, 0.25 for DRG 467, 0.26 for DRG 468, 0.41 for DRG 469, and 0.09 for DRG 470, with no notable correlation between Medicare reimbursement and chargemaster listing price across any DRG (Table 4, Supplemental Table 1, http://links.lww.com/JG9/A298).

**Table 4:** Comparison of the Mean Medicare Reimbursement to Hospitals and Mean Chargemaster Price

DRG Code	Mean Medicare Reimbursement to Hospitals (USD)	Mean Chargemaster Price (USD)	Percentage of Chargemaster Price Reimbursed by Medicare	R-Squared Correlation Between Medicare Reimbursement and Chargemaster Price; *P*
466	$36,122.61	$221,927	15.16%	0.15; *P* = 0.52
467	$21,921.77	$117,290	14.82%	0.25; *P* = 0.28
468	$17,174.16	$90,966	14.70%	0.26; *P* = 0.27
469	$19,733.56	$128,290	12.79%	0.41; *P* = 0.07
470	$11,828.10	$68,016	13.90%	0.09; *P* = 0.71

DRG = diagnosis-related group

## Discussion

Our study demonstrates a substantial variability of hospital chargemaster prices for total joint arthroplasty procedures among top orthopaedic hospitals in the United States. Chargemaster price variations for arthroplasty procedures among the top 20 ranked orthopaedic hospitals had a 3.2- and 3.8-fold difference between the 10th and 90th percentiles of listing amounts. This wide price variation was similar, but higher, to the national relative price variation found in other studies, where the price variation according to the national was reported to be 229% for knee arthroplasty from 2007 to 2011.^18,19^ Furthermore, the listed charges are not correlated with the Medicare reimbursement that the institutions included in our study are receiving, despite Medicare being the most common payor of elective total joint arthroplasty in the United States. Given the immense level of price variation for the same procedures at different hospitals with little correlation with the cost of living, local median income, or Medicare reimbursement, it is unlikely that the use of chargemasters is fulfilling their intended purpose of informing price transparency for patients. Large variations between similar-quality hospitals, even between hospitals located in the same state, make chargemaster prices an unreliable resource for consumers.^19^ Of note, only 18 of the 20 hospitals had accessible chargemasters, possibly because hospitals are reluctant to share their financial information because of insurance negotiation leverage.^20^ In addition, the penalty for not having an accessible chargemaster is enforced by a written notice and a $300 dollar per day penalty, which for a large hospital is largely inconsequential.^20^

The large variability in the chargemaster price between major hospitals points to a larger issue: the price of a service listed on a chargemaster provides very small benefit to the average patient in need of a total joint arthroplasty. For a patient to benefit from healthcare cost transparency, they should be able to accurately predict and plan for the expense of total joint arthroplasty. Accordingly, in the current system, the patient would need to be familiar with the various components of the surgery being billed, such as physician charges, other provider charges, and facility fees.^12^ However, the prices that hospitals list on the chargemaster are seldomly the amount of money spent by patients because every insurance plan is widely variable with different negotiations, out-of-pocket expenses, and coverage. For example, a study of 34 Medicare patients undergoing total hip arthroplasty (THA) found that even among the same insurance provider, the difference in out-of-pocket costs ranged by a factor of 18.5x between the lowest out-of-pocket cost and the highest out-of-pocket cost.^21^ Furthermore, even if these prices were known, patients could unknowingly receive care from providers out of network and receive surprise bills far greater than their total out-of-pocket maximum of their health insurance. Recent estimates claim that one in six patients undergoing elective orthopaedic surgery is at risk of a potential surprise bill, which, surprisingly, is comparable with unplanned, emergency services provided in the emergency department.^22-24^ Chargemaster pricing could potentially help make a rough estimation of a procedure's cost if a patient has no insurance, but an average patient with insurance will pay a vastly different and often pre-emptively unknown sum after insurance.

This lack of healthcare cost transparency is costly to patients because elective arthroplasty is considered a “shoppable service,” where patients should be able to pick and choose where to get an arthroplasty based on cost, ratings, level of health care, expertise, location, and other factors. A study performed by the Health Care Cost Institute estimated that individuals with employee-sponsored insurance in the United States spent $524 billion on health care in 2016, with 43% of that amount spent on medical services that could be classified as “shoppable services.”^25^ Increased price transparency is thought to influence market dynamics for elective procedures and encourage competition. Such competition may result in more reasonable prices for elective healthcare services such as arthroplasty, as current financial pressures incentivize high pricing to aid in insurance reimbursement negotiations and to maximize charity care tax deductions when a medical service is provided for free.^20^

Although chargemasters have largely failed to increase price transparency for patients, our analysis of chargemasters has allowed us to discover large price variation among the top ranked hospitals included in this analysis, which is peculiar because these highly ranked orthopaedic hospitals likely provide similar high-quality treatment. Similarly, there is a limited correlation of chargemaster pricing and the cost of living or wage index of the city in which the hospital is located. This is interesting because national insurers, such as Medicare and Medicaid, break down reimbursement into a labor component and a nonlabor component, with each component designed to account for geographical variations in the cost.^26^ An analysis from 2013 examining regional differences in healthcare costs found that the Pacific region, consisting of Washington, Oregon, California, Alaska, and Hawaii, had notably higher inpatient charges per stay at 37% higher than the national average.^27^ Although our analysis contained five hospitals in this Pacific region with the increased cost of living and increased median income, our analysis demonstrated that these factors were not strong predictors of variation in chargemaster pricing for total joint arthroplasty.

The discussion of price transparency continues to increase in relevance with the recent implementation of the Hospital Price Transparency Rule as issued by the Department of Health and Human Services.^28^ The rule, which went into effect on January 1, 2021, adds clarity and increased transparency regarding the standard and public release of standard charges across all hospitals in the United States. Before this ruling, hospitals could comply with the aforementioned chargemaster requirement simply by posting any arbitrary charge somewhere on their website for the services they offer. However, the new ruling clarifies that hospitals now must meet a minimum standard with such reporting, which includes the display of at least 300 “shoppable services,” in which the charges for such procedures are listed in “plain language and a consumer-friendly display.” In addition, the rule now requires that for these services the hospital also provide a discounted cash price for uninsured or self-pay patients, as well as the public release of payer-specific negotiated charges.

The public release of payer-specific negotiated charges is a substantial change because the negotiations between hospitals and private insurers have historically been private. The private nature of these pricing negotiations contributed to the general uninformed state of pricing for medical services among patients in the United States. It remains to be seen what the impact of this ruling will have on pricing, negotiations, and consumer knowledge within health care. It is clear that for chargemasters to improve healthcare transparency for patients and control healthcare spending, chargemasters need to be revamped synergistically with the creation of new innovative healthcare policy to protect patients, hospitals, healthcare workers, employers, and insurance companies.

Our study has several limitations. First, given the nature of our study, our chargemaster data come from publicly available and individual hospital websites. Given the variability between websites and the variability in the display of chargemaster data, it is possible that collected data may not be comprehensive of all pricing information that is offered publicly by each hospital. However, the authors believe this to be reflective of the variability between the patient experience and the current reality of attempting to collect these pricing data. Finally, we selected the top 20 US orthopaedic hospitals according to the US News and World Report to control for quality of medical care; however, these hospitals may not be reflective of the global arthroplasty market in the United States. Notwithstanding, our study clearly identifies the impressive variability of hospital chargemaster prices.

## Conclusion

The published cost of DRG codes in arthroplasty is widely variable among the top 20 US orthopaedic hospitals, with the same procedure varying in costs by hundreds of thousands of dollars between hospitals of similar quality, with little correlation with Medicare reimbursement, cost of living, or median income of the location of the hospital. Further analysis is needed to understand how hospitals determine chargemaster costs, and how these costs correlate with the actual out-of-pocket cost. Policymakers should be aware of these trends because we attempt to increase healthcare transparency in the United States and assess the utility of chargemasters as a method to improve transparency.

## Supplementary Material

SUPPLEMENTARY MATERIAL
