# Supplementary material for: Large Variation in Listed Chargemaster Price for Total Joint Arthroplasty Among Top Orthopaedic Hospitals in the United States
Source: J Am Acad Orthop Surg Glob Res Rev. 2023 Sep 7;7(9):e23.00052. doi: 10.5435/JAAOSGlobal-D-23-00052 (PMC10489520; doi:10.5435/JAAOSGlobal-D-23-00052)
Supplement: SUPPLEMENTARY MATERIAL [file jagrr-7-e23.00052-s001.docx]

| Hospital | 466 - REVISION OF HIP OR KNEE REPLACEMENT W/ MCC | 467 - REVISION OF HIP OR KNEE REPLACEMENT W/ CC | 468 - REVISION OF HIP OR KNEE REPLACEMENT W/O CC/MCC | 469 - MAJOR HIP AND KNEE JOINT REPLACEMENT OR REATTACHMENT OF LOWER EXTREMITY W/ MCC OR TOTAL ANKLE REPLACEMENT | 470 - MAJOR HIP AND KNEE JOINT REPLACEMENT OR REATTACHMENT OF LOWER EXTREMITY W/O MCC |
| --- | --- | --- | --- | --- | --- |
| 1 | $107,582 | $68,817 | $58,967 | $74,588 | $39,927 |
| 2 | $116,930 | $72,944 | $60,653 | $77,645 | $40,008 |
| 3 | $132,501 | $86,588 | $65,160 | $79,809 | $49,478 |
| 4 | $132,929 | $92,639 | $69,569 | $97,363 | $53,366 |
| 5 | $168,684 | $99,085 | $71,580 | $99,530 | $54,677 |
| 6 | $176,393 | $102,961 | $72,401 | $101,791 | $54,850 |
| 7 | $184,431 | $112,298 | $74,070 | $123,207 | $59,680 |
| 8 | $197,687 | $117,181 | $86,171 | $126,285 | $67,481 |
| 9 | $221,927 | $117,290 | $90,848 | $128,290 | $68,016 |
| 10 | $249,736 | $122,401 | $91,085 | $132,602 | $68,704 |
| 11 | $250,810 | $133,493 | $93,807 | $145,811 | $69,316 |
| 12 | $252,672 | $133,952 | $119,622 | $147,130 | $78,599 |
| 13 | $261,619 | $161,095 | $132,078 | $175,068 | $91,185 |
| 14 | $313,056 | $183,688 | $141,861 | $201,797 | $104,179 |
| 15 | $384,867 | $275,324 | $156,863 | $264,500 | $160,440 |
| 16 | $427,423 | $317,765 | $224,416 | $321,955 | $191,787 |
| 17 | $472,517 | $317,806 | $246,364 | $324,985 | $195,264 |
| 18 | x | x | $247,715 | x | x |
| **Mean** | **$238,339** | **$147,960** | **$116,846** | **$154,256** | **$85,115** |
| **Median** | **$221,927** | **$117,290** | **$90,966** | **$128,290** | **$68,016** |
